# Experimental comparative study of a novel drug-eluting arteriovenous graft in a sheep model

**DOI:** 10.3389/fcvm.2024.1341154

**Published:** 2024-02-26

**Authors:** Sara Schødt Riber, Lene Langhoff Clausen, Marie Dahl, Lars Peter Schødt Riber, Thomas Emil Andersen, Jes S. Lindholt

**Affiliations:** ^1^ Department of Cardiothoracic and Vascular Surgery, Odense University Hospital, Odense, Denmark; ^2^ Department of Clinical Research, University of Southern Denmark, Odense, Denmark; ^3^ Elite Research Centre of Individualized Medicine in Arterial Disease (CIMA), Odense University Hospital, Odense, Denmark; ^4^ Vascular Research Unit, Department of Vascular Surgery, Viborg Regional Hospital, Viborg, Denmark; ^5^ Department of Clinical Medicine, Aarhus University, Aarhus, Denmark; ^6^ Department of Cardiovascular and Renal Research, Institute of Molecular Medicine, University of Southern Denmark, Odense, Denmark

**Keywords:** experimental animal models, vascular surgical procedures, surgical, expanded PTFE, innovativeness

## Abstract

**Background:**

Arteriovenous (AV) grafts often develop severe complications of stenosis due to neointimal proliferation that occurs either at the venous anastomosis site or at the outflow receiving vein. This study compares primary patency during 12 months of follow up for a new experimental Biomodics^©^ interpenetrating polymer network (IPN) drug-eluting graft prototype with state-of-the-art GORE® ACUSEAL (ACUSEAL) in an AV graft model in sheep.

**Methods and results:**

An end-to-end bypass from the common carotid artery to the jugularis vein was performed bilaterally in 12 sheep. The usage of ACUSEAL or the IPN, both 6.0 mm in diameter, was determined via randomization. The sheep were followed up every 4 weeks with ultrasonic duplex scanning to determine patency; the experienced observer was blinded to the randomization. One sheep died after 11 days, and the final sample accordingly consisted of 11 animals. When comparing neointimal hyperplasia after 12 months in the two grafts, Fisher's exact test showed a significant difference with none out of 11 in the IPN grafts and 9 out of 11 in the ACUSEAL graft (*p* < 0.001). However, the Biomodics^©^ IPN exhibited severe deterioration over time.

**Conclusions:**

Almost all of the grafts occluded during the 12 months of follow up. Although the zwitterion-bounded interpenetrating drug eluting polymer network showed signs to impair neointimal hyperplasia and thrombosis, age-related degeneration hindered demonstrating a potential improvement in patency.

## 1 Introduction

The incidence of chronic kidney disease (CKD) is increasing dramatically, and the cost of treatment represents an enormous burden on global healthcare systems (1). This disease is a leading cause of morbidity and mortality worldwide; it ranks 12th on the list of global causes of death (2). In 2017, 1.2 million people died from kidney failure, an increase of 41.5% since 1990 (2).

Treatment options for advanced CKD include either hemodialysis (HD) or kidney transplantation from a compatible donor. For end-stage CKD patients deemed to be suitable transplantation recipients, kidney transplantation is preferable—the associated five-year survival rate is over 80% for transplantation (3) compared with 35% for patients on HD. Unfortunately, an acute global shortage of kidney donors means that most end-stage CKD patients rely on HD for treatment: in 2015, over 31-times more CKD patients were treated with HD than transplants (4, 5).

Patients requiring long-term HD are preferably given an arterial-vein fistula (AVF) connecting a vein to an artery on the upper arm or forearm for easy, safe, and durable access. However, up to half of all AVFs do not “mature” properly. Furthermore, due to repeated needs for AVF, the ability to create novel AVFs is generally exhausted over time. The second choice for HD access is an artificial arteriovenous (AV) graft that connects the vein and artery in the arm or leg in a looped tube. Arteriovenous grafts are often made from expanded polytetrafluoroethylene (ePTFE) and typically develop neointimal hyperplasia (NIH) that can result in thrombosis, graft infections, and/or bleeding due to numerous punctures. All of these issues can in turn limit the long-term viability of grafts (6). A meta-analysis conducted in 2020 concluded that the primary patency for ePTFE AV grafts after 12 months is 41%; secondary patency is 70% (7). Therefore, improving the materials used for AV grafts has the ability to improve quality of life for CKD patients on permanent HD.

Paclitaxel (PTX) has previously been shown to inhibit smooth muscle cell proliferation and migration and prevent NIH (8). Today, it is widely used for arterial stents and drug-eluting balloons. Recent research also suggest that one encounter higher primary patency rates when stenoses of the anastomotic sites of an AV graft are treated with PTX-eluting balloons rather than plain balloons (9). However, drug-eluting vascular grafts are not currently available for clinical use.

A new AV graft composed of a Biomodics^©^ interpenetrating polymer network (IPN) material is currently under development that consists of silicone elastomer and zwitterionic hydrogels (10–14). Their hypothesis is that zwitterionic polymer material, containing 4-(1-vinyl-3-imidazolio) butanesulfonate, mimics the chemistry of the inner surface of native blood vessels; the innate immune and coagulation system does not recognize it as a foreign body. As a result, this material is characterized by improved biocompatibility and is less likely to activate the complement system, which can trigger coagulation. The multiporous IPN polymer also possesses drug loading and release properties that inhibit NIH and infections (15).

The aim of this study was to investigate whether the Biomodics^©^ IPN graft could be superior in terms of producing less NIH and thereby have longer open AV-grafts than the known heparin-coated GORE® ACUSEAL (ACUSEAL).

## 2 Materials and methods

### 2.1 Design

The study's design and experimental animal model was based on a previous study published by Liisberg et al. that used a prospective, paired, randomized study design in a sheep animal model (16).

We compared the new IPN graft loaded with PTX with the self-sealing ACUSEAL in a paired sheep carotid-jugularis model with 12 months of follow up. The two types of grafts were randomly implanted on the right or the left side of the necks of 12 sheep, according to a fixed randomization key. The postoperative ultrasonographic observer of the graft function (MD) was blinded to the graft allocation and remained blinded until the data were analyzed. Graft failure, defined as any state other than an intact open and uninfected graft, was used as the primary endpoint. The study protocol was approved by the Danish animal experiments inspectorate (no: 2016-15-0201-01089).

### 2.2 Statistical analyses

The sample size was derived from power calculation with 0.8 power, a 0.05 significance level, and a 0.2 correlation coefficient for graft failure between the paired AV grafts. We expected a reduced graft occlusion rate ranging from 65% for ACUSEAL grafts (17) to only 15% for IPN grafts based on in-vitro examinations. According to Dupont's method, a sample of 11 sheep was required. Twelve sheep were accordingly included to allow for drop out.

The patency of each graft was recorded in days, and the difference of NIH between the grafts was analyzed using Fisher exact test. Additionally, survival analysis was graphed using a Kaplan-Meier plot. IBM SPSS Statistics (version 28.0.1) (IBM®, USA) was used for all of the analyses and illustrations.

### 2.3 Graft materials

The IPN graft is produced in supercritical carbon dioxide (scCO_2_) and comprises an IPN of extruded silicone elastomer (Nusil MED-4027, 5.0 mm inner diameter × 0.75 mm wall thickness × 500 mm length) as the host polymer (13). It has a cross-linked random terpolymer of 2-hydroxyethyl methacrylate (HEMA), poly (ethylene glycol) methyl ether acrylate (PEGMEA), and 3-(1-Vinyl-3-imidazolio) butanesulfonate (VIBS) [poly(HEMA-co-PEGMEA-co-VIBS)] as the guest polymer. The IPN procedure makes the graft material swell in size, which explains the final inner diameter of 6.0 mm (13). The inner lumen of the graft is subsequently loaded with 6 mg/ml PTX (“Fresenius Kabi”) in a 1:1 mixture of castor oil and ethanol for 2.5 h.

The ACUSEAL graft has also an inner diameter of 6.0 mm and a tri-layer design that allows for early cannulation because internal epithelization is not required prior to use. The inner lumen layer is ePTFE with a coating of Carmeda's Bioactive Surface (CBAS) covalent bonded heparin. The middle layer is made of a silicone elastomer that self-seals after puncture with a cannula. The outer layer is ePTFE (18).

### 2.4 Animal model

Wild-type crossbreed sheep that incorporated Texel bloodlines. We limited our sample to female sheep, which are usually more docile than males, to facilitate handling during postoperative care and examination.

### 2.5 Anesthetic

Food and water were withheld from the sheep for 36 and 12 h, respectively, prior to surgery. Anesthesia induced with an intramuscular injection of ketamine (5 mg/kg) and diazepam (0.25 mg/kg). During surgery, the sheep were anesthetized with a combination of propofol (10 mg/kg/h) and fentanyl (Haldid 25 µg/kg/h).

### 2.6 Implantation of the graft

We made a cut to expose the carotid artery and internal jugular vein using a standard open surgical technique. Roughly 5 cm of the artery was exposed; the precise length depended on the muscular configuration of the sheep and was selected to avoid damaging muscles and/or the Vagus nerve. Before the carotid artery was clamped, 5,000 IU Heparin was administered intravenously to prevent clotting during anastomosis. The ends of the graft were cut at a 45-degree angle and anastomosed in an end-to-side fashion using 5–0 Prolene® (B Braun, Germany) first to the carotid artery and next to the jugular vein. After confirming the patency of the anastomosis and the grafts themselves, we ensured hemostasis. The wound was closed in two layers using self-absorbing 2-0 Vicryl® (Ethicon Inc., USA) in the subcutis and PDS® in the skin (Ethicon Inc.). After the wound was closed, it was sprayed with fluent banding OPSITE® (Smith & Nephew A/S, USA) to inhibit bacterial contamination.

### 2.7 Follow up

The sheep were maintained in the animal facility department for optimal care during the first week following surgery. They were then moved to pasture. Once every four weeks, they returned to the animal facility for one day to undergo an ultrasonographic control scan. This follow up was repeated for one year.

All of the sheep were color duplex scanned using a portable GE® Logiq E ultrasound device with a linear probe; the animals were mildly sedated with propofol. All of the grafts were scanned every month for occlusion and thrill. This procedure was repeated regardless of earlier findings to ensure that the blinded investigator performed the scans identically each time.

### 2.8 Termination and post-analyses

After the last scan was performed one year postoperatively, the sheep were euthanized with a lethal intravenous injection of phenobarbital (100 mg/kg). After termination, the grafts were carefully extracted from the sheep and macroscopically evaluated by three surgeons in preparation for histology and tension tests.

For histology analysis the anastomosis and middle section of each graft was examined for hyperplasia using Masson's trichrome and Weigert staining. Tension tests were also performed in two axes using a 50 N Microtest tensile stage (Deben UK Limited, UK).

## 3 Results

### 3.1 Surgical outcomes

Of the 12 sheep used in this study, the animals' median age was 3.4 years (range: 3.4–4.4 years). The animals' mean weight was 61.6 kg (range: 53.2–73.4 kg).

All 12 sheep underwent bilateral AV grafting, but one sheep had to be terminated at day 11 due to its poor respiratory condition. The remaining 11 sheep survived to the first postoperative month; one sheep required re-suturing of its skin two weeks after surgery. All 11 sheep were transferred to pastures between the second and third postoperative week, but one sheep died on day 70 and another died on day 80. Blinding for these animals was then broken; both sheep had already been diagnosed with an occluded bilateral graft from the duplex scans at 2 months. They had accordingly already fulfilled the end-point criteria prior to their death, and they are therefore included in our results.

**Figure 1:**
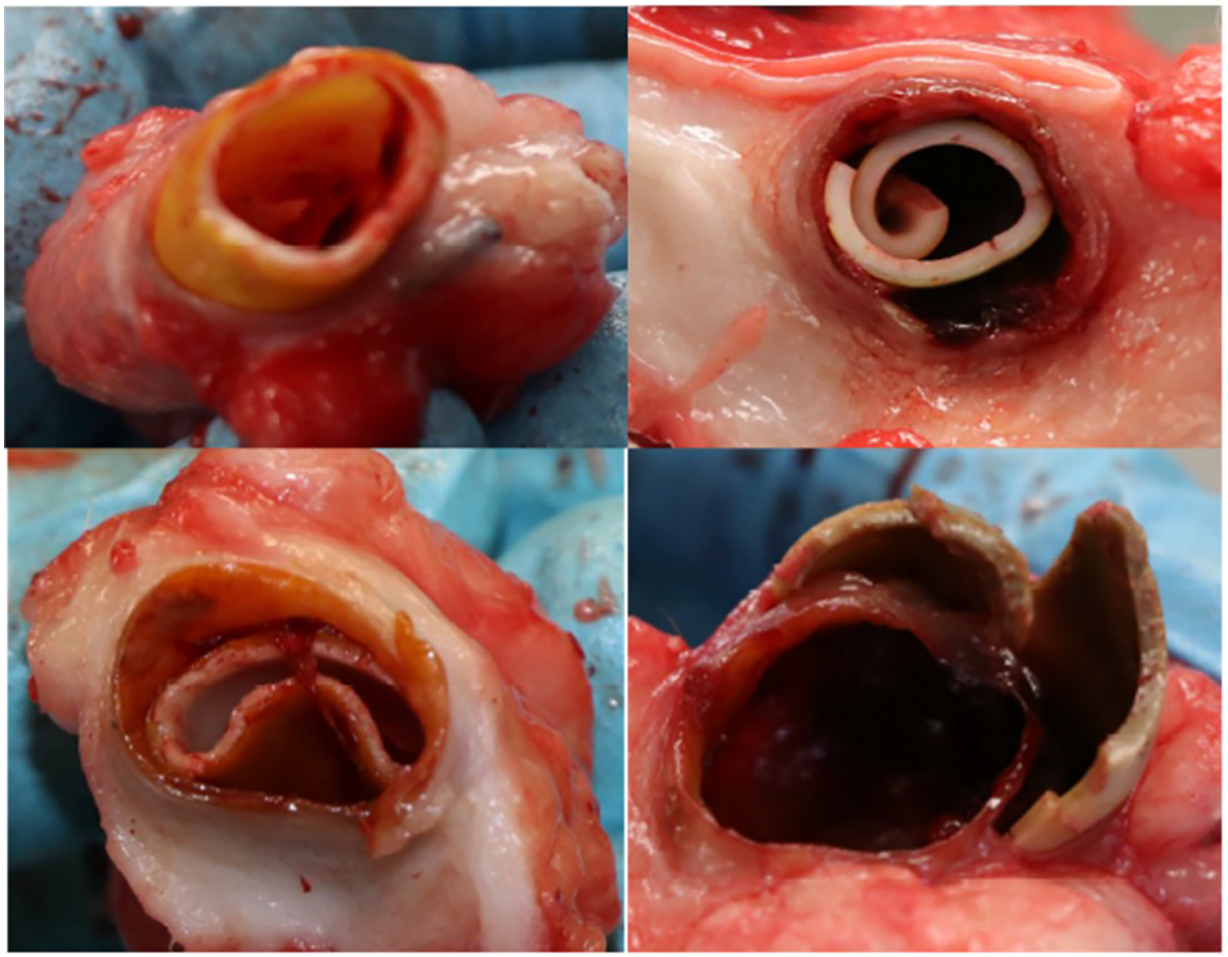
Biomodics IPN graft at the autopsy.

**Figure 2:**
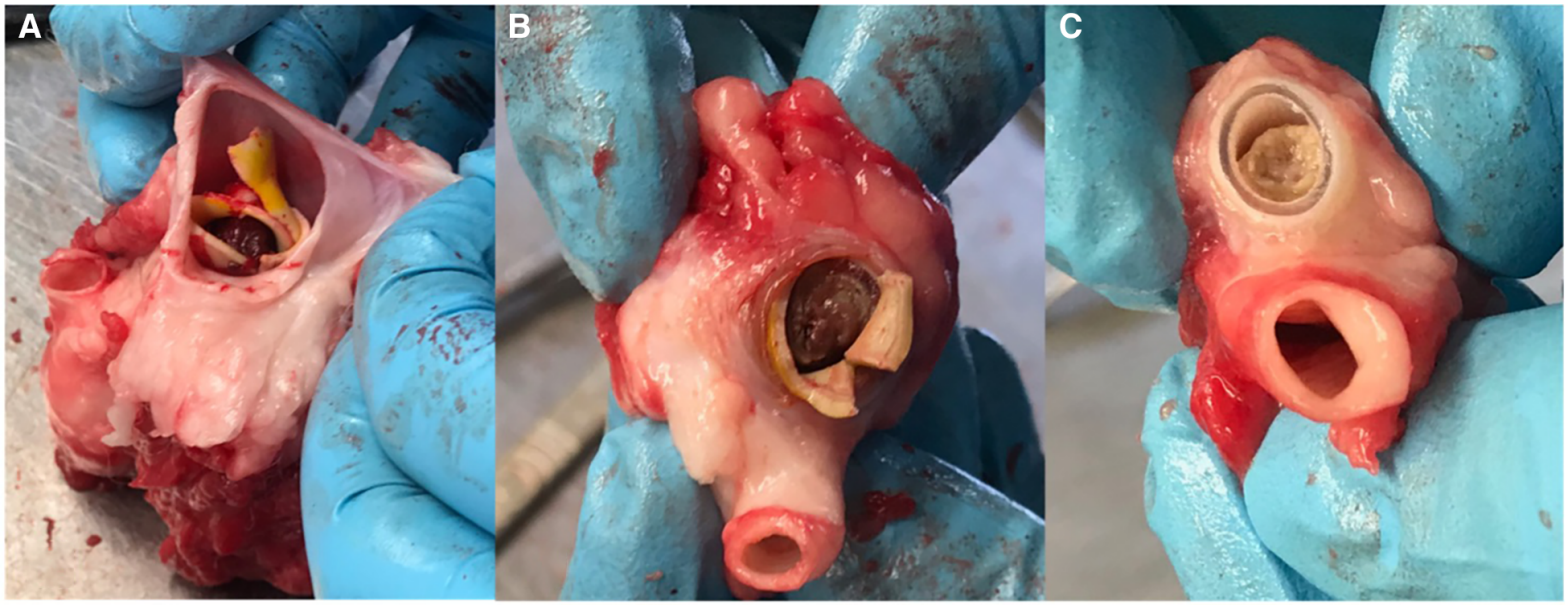
(**A**) The fibrotic tissue, which surrounded the IPN graft and allowed continued blood flow. (**B**) A thrombotic IPN graft at autopsy. (**C**) An occluded Acuseal at autopsy.

After 12 months of follow up, the last ultrasonographic control scan indicated that two ACUSEAL grafts (18%) and six IPN grafts (55%) still had patent flow. However, after the animals were euthanized and the ACUSEAL and IPN grafts were exposed and removed for further examination, it became clear that the IPN material had deteriorated in all of the sheep except for one. We furthermore noted that the IPN material broke apart with even light manipulation (Figure 1). No such deterioration was observed in the ACUSEAL grafts. The fibrotic tissue surrounding the IPNs had created a channel that maintained the flow noted on the duplex scans, despite the deterioration of the IPN. However, all the occluded ACUSEAL grafts had developed NIH throughout the entire length of the graft. Therefore, no initiation site of NIH couldn't be detected. Figures 2A,B shows deteriorated IPN graft and thrombus formation, while Figure 2C shows NIH in the ACUSEAL graft.

We mistakenly interpretated that signal as a primary patent graft. Consequently, we were forced to conduct post-hoc analyses.

### 3.2 Post-hoc analyses

**Figure 3:**
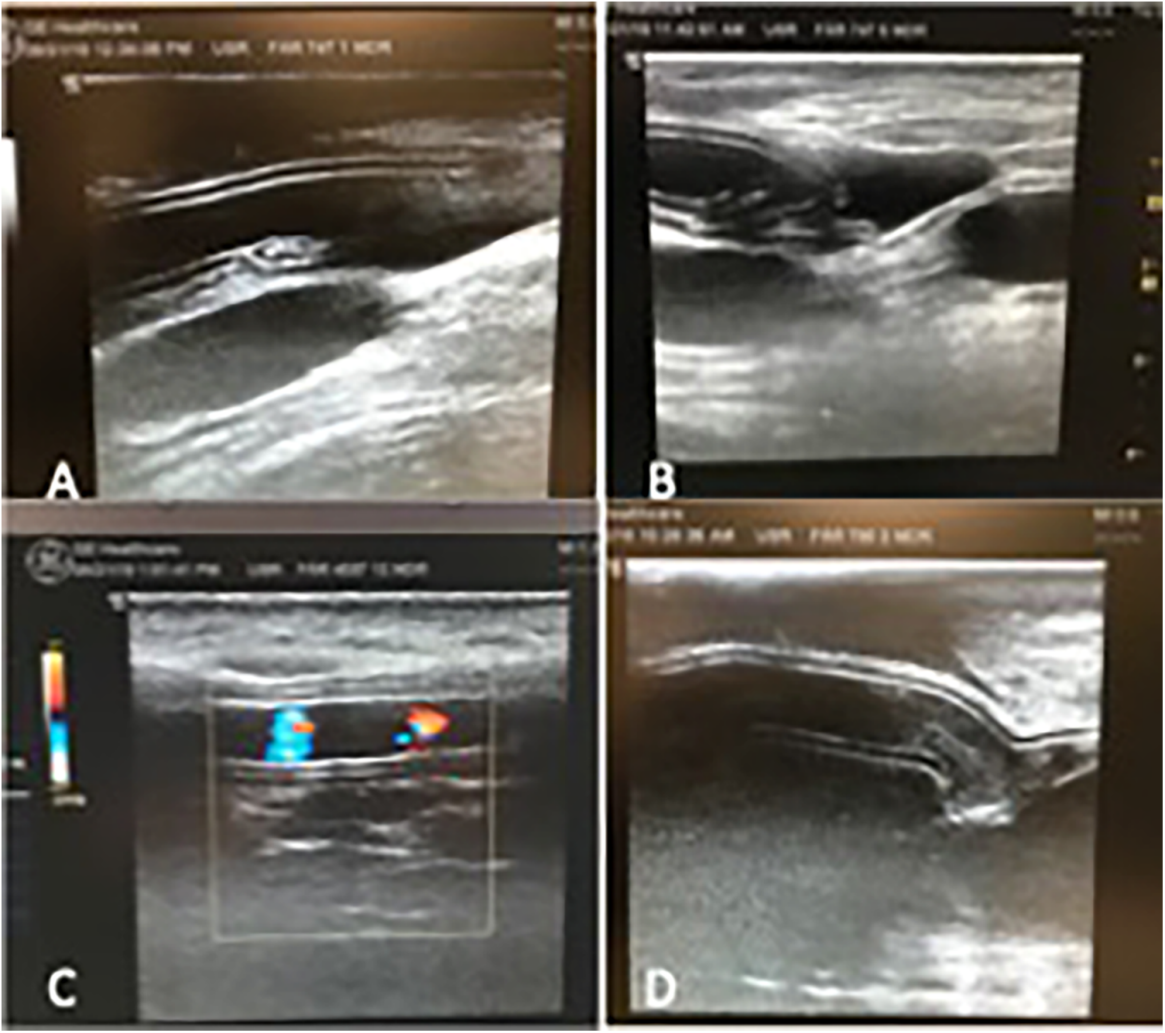
(**A**) Open IPN graft before material failure. (**B**) IPN graft same as (**A**), but now degenerated with material failure. (**C**) Open ACUSEAL graft (**D**) occluded ACUSEAL.

**Figure 4:**
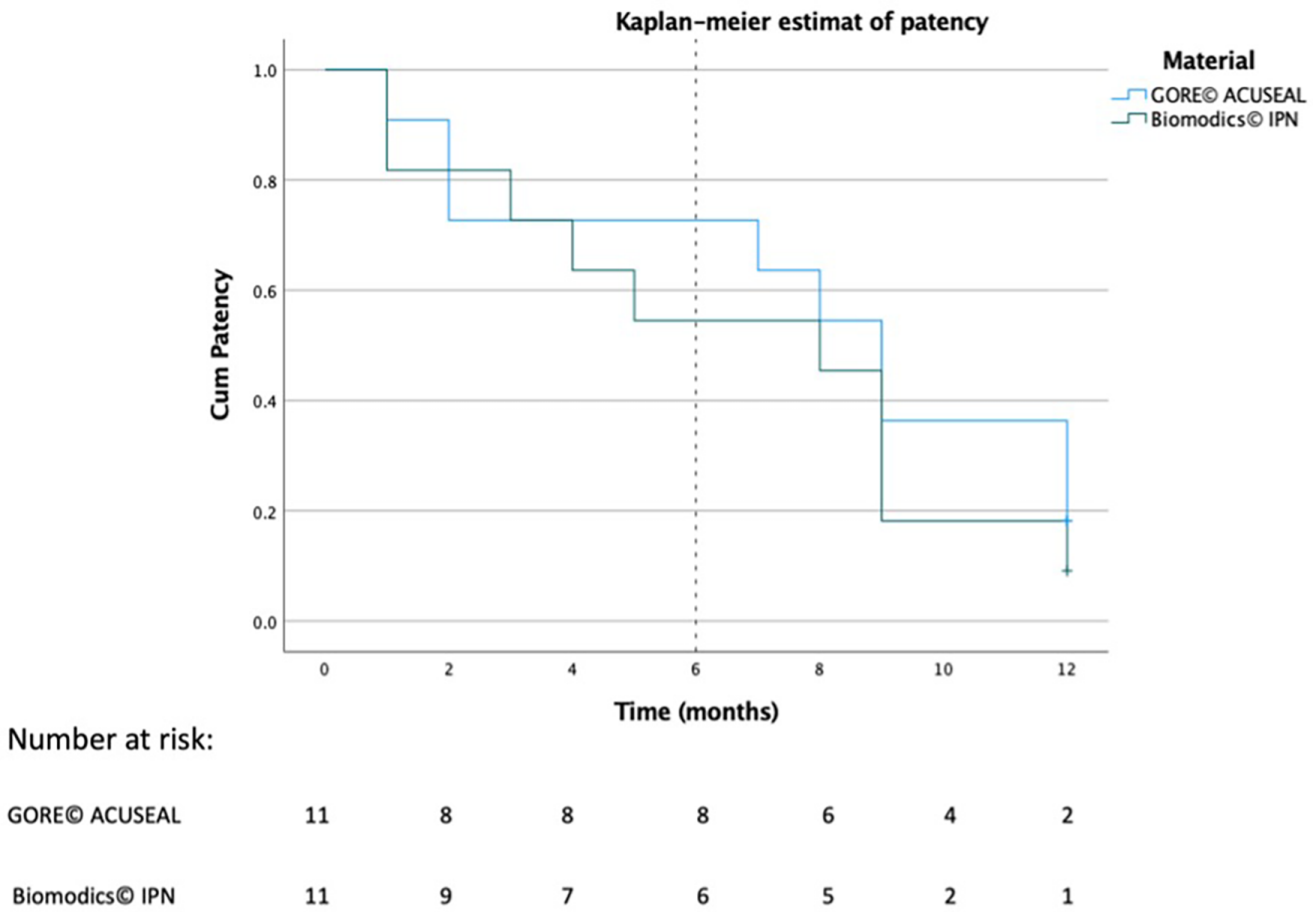
Kaplan–Meier plots of adjusted patency.

Two independent observers reviewed the ultrasound scans and detected a loose flap on the graft line in two of the IPN grafts at the site of the anastomosis beginning in week 16. (However, flow was still maintained in the grafts.) These grafts were therefore classified as graft failures beginning in week 16. Examples of patent grafts free of deterioration and occlusion are illustrated in Figure 3. After 12 months of follow up, two of the ACUSEAL grafts (18%) and one of the IPN grafts (9%) were failure-free (Table 1; Figure 4). In regard to neointimal hyperplasia, Fisher's exact test showed a significant difference with none out of 11 in the IPN grafts and 9 out of 11 in the ACUSEAL grafts (*p* < 0.001).

**Table 1:** Data from 12 months duplex ultrasonography control. Data are given as number of open grafts, with percentages given in parentheses.

Month	0	1	2	3	4	5	6	7	8	9	10	11	12
ACUSEAL	11 (100)	10 (91)	8 (73)	8 (73)	8 (73)	8 (73)	8 (73)	7 (64)	6 (55)	5 (45)	4 (36)	4 (36)	2 (18)
IPN	11 (100)	9 (82)	9 (82)	8 (73)	7 (64)	6 (55)	6 (55)	6 (55)	5 (45)	2 (18)	2 (18)	2 (18)	1 (9)

### 3.3 Tensile tests

The tensile strengths of the undeteriorated IPN grafts, as well as a similar unimplanted IPN graft and one explanted ACUSEAL graft, were analyzed ex vivo. The tests were conducted until failure to determine the strain breakage limit. We found that the IPN material had lost significant elasticity and tensile strength over one year (93%); the ACUSEAL material was still unbreakable at the maximum force after one year (Table 2).

**Table 2:** Strength of the paclitaxel loaded biomodics® IPN before and after one year implantation, and the GORE^©^ ACUSEAL by uniaxial tensometry.

Material	Circum stress (kPa)
Biomodics® IPN with Paclitaxel before implantation^a^	4,062
Biomodics® IPN with Paclitaxel—after one year implantation in sheep^b^	278
GORE^©^ ACUSEAL	>14,000

^a^ Mean value of 4 samples.

^b^ Only one sample of the graft material from sheep could be tested correctly. The rest were so fragile, that they busted at the clamp site during stretch and therefore had to be excluded.

### 3.4 Histology

**Figure 5:**
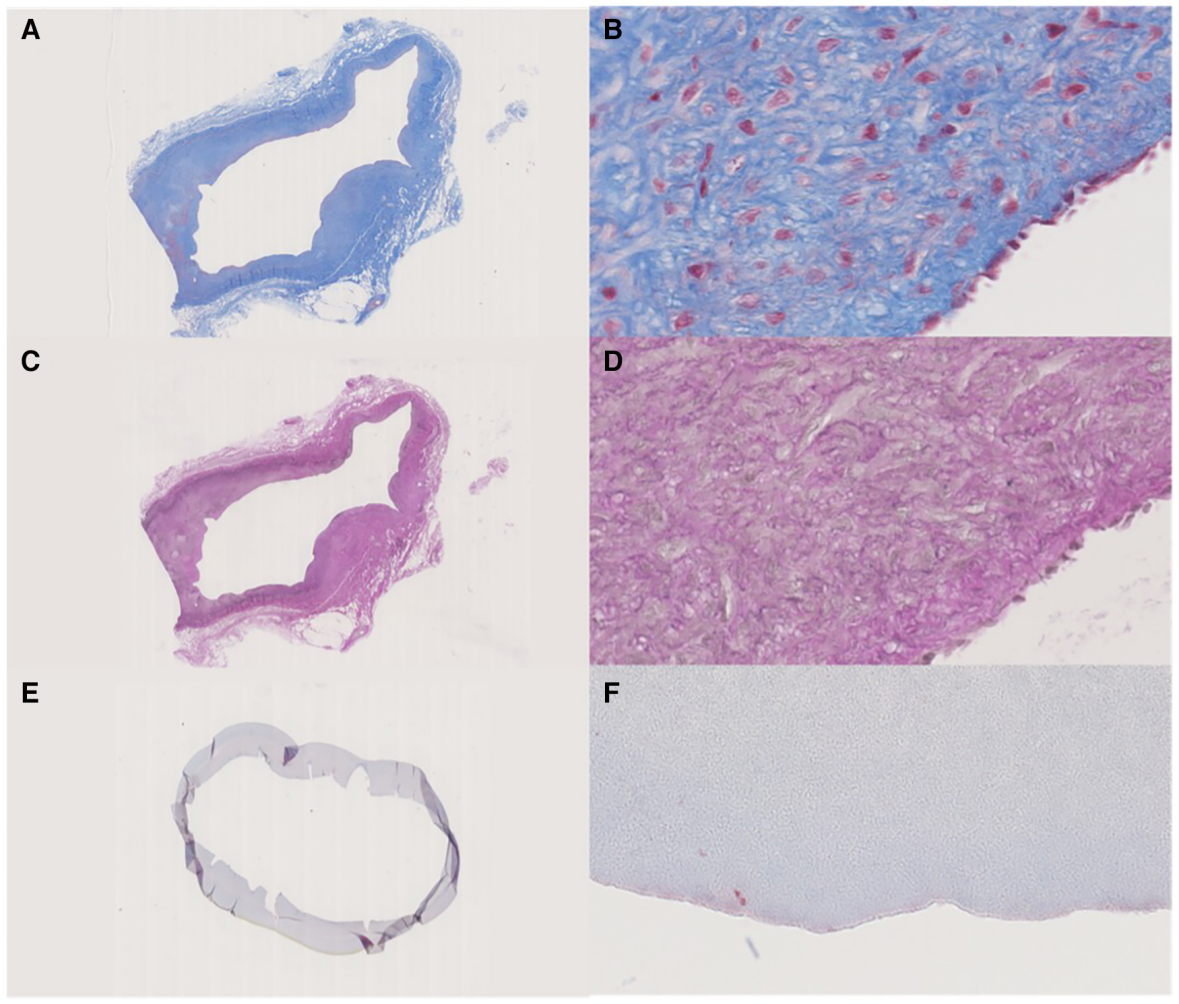
(**A**,**B**) Masson's trichrome stain, the fibrotic tissue that encapsulated the IPN graft. (**A**) Overview, (**B**) lumen site 40x. (**C**,**D**) Weigert's elastin stain of the fibrotic tissue that encapsulated the IPN graft. (**C**) Overview, (**D**) lumen site 40x. (**E**,**F**) Masson's trichrome stain, Cracked IPN graft, but with no ingrown cells in the lumen. (**E**) Overview, (**F**) lumen site 40x.

The tissue specimens retrieved after autopsy were very fragile. The IPN had separated from the surrounding tissue, but no NIH was observed (Figure 5). The ACUSEAL graft was too hard to cut after casting, which rendered histological sample staining impossible.

## 4 Discussion

This study showed that the failure-free rates of IPN and ACUSEAL grafts after one year were similar, since failure due to biodegradable IPN grafts happened at the same speed as NIH in the ACUSEAL graft. No NIH was seen at any time in the IPN grafts.

One sheep died from respiratory distress at day 11. Mortality is a known risk when performing sheep interventions, which is why we designed the study to include one more sheep than the power calculation suggested. Two additional sheep that were found dead in the field were autopsied, but we did not determine the cause of death. However, the grafts in these two deceased sheep had already failed based on the scanning conducted in the second month. For that reason, these sheep were included in the final analyses.

We found that a higher fraction of implanted IPN grafts sustained a patent flow at the UL scans throughout the trial compared with the ACUSEAL grafts. However, the sustained flow was due to formation of a tubular fibrotic sheath surrounding the IPN, since the IPN itself almost had completely deteriorated (Figure 2A). Tensometry of the single undisintegrated IPN graft revealed a loss of 93% of its strength (Table 2) after one year of implantation.

Castor oil, the material in which the PTX was dissolved, has been reported to leach the plasticizer DEHP [di- (2-ethylhexyl) phthalate] from polyvinyl (PVC)-containing solution bags and administration sets (19). However, IPN does not contain DEHP; it instead contains polyethylene glycol (PEG). Therefore, it is unclear why castor oil would weaken IPN material. It is more likely that oxidation of the IPN graft occurred (20, 21). These considerations and more are undergoing further investigation by the consortium behind the European H2020 project TELEGRAFT (20).

No NIH was found at any of the IPN sites upon autopsy (Figure 2B). Some of the occluded IPNs had clotted blood in the inner lumen. However, NIH accompanied all of the occluded ACUSEAL grafts (Figure 2C). This finding indicates that the PTX-eluting IPNs have the potential to prevent NIH.

The primary graft failures could have been due to poorly conducted anastomotic surgeries; the procedures were performed in a different environment than surgical procedures conducted in humans. However, all of the surgeries were performed by experienced vascular surgeons; if the occlusions were due to poorly conducted surgeries, we should have expected to see more occlusions within the first postoperative month. We note that animal models always pose challenges.

Both the ACUSEAL and IPN grafts exhibited similar levels of flow during the trial period. The ACUSEAL grafts occluded in the end and the IPN grafts deteriorated, but the IPN material appeared to withstand clotting and maintain flow. However, that latter finding could be explained by a fibrotic channel that had formed around the graft. Our results suggest that IPN grafts could benefit from improved strength and durability, which could perhaps be obtained by changing the interpenetrating procedure to avoid the use of PEGylated acrylate and instead use a PEGylated acrylamide, which is more resistant to oxidation and hydrolysis.

The cause of the fibrosis surrounding the IPN grafts also needs to be identified and addressed; silicone is a well-accepted material that is often used for transplants. Fibrosis can also potentially affect the mechanical stability of a graft and pose a challenge for reoperation to ensure secondary patency. We suspect that the PTX loaded into the graft luminal surface diffused via the multiporous interpenetrating network to the outer surface and resulted in tissue damage and fibrosis. In a not yet published PTX study from our group, wound complications were common, which supports our hypothesis. This problem could be solved by adding a barrier layer on the outer surface of the graft. Additional investigation and development is necessary to solve these problems and produce a device resistant to thrombosis, deterioration, and fibrosis.

### 4.1 Study limitations

This study is subject to several limitations. To begin with, we had to rely on retrospective evaluations of the graft failures; we were unable to identify deterioration prior to the end of the study. We were accordingly unable to determine the exact time of material deterioration. That situation raises the risk of information bias; we only had a limited amount of data for the post-hoc evaluation. As a result, we might have overestimated the performance of the IPN. However, this situation would not change our conclusions. Given that deterioration of an IPN graft is unacceptable in humans, additional work is necessary to develop next-generation vascular grafts.

## 5 Conclusion

The interpenetrating polymer network grafts examined in our study had poor biostability and fibrotic side effects which cannot be tolerated in humans. The future of IPN as a graft material is dependent on further additional research to alter this material to become bio-convertible, if its promising properties in terms of resistance against NIH and thrombosis should benefit patients.

## Data Availability

The raw data supporting the conclusions of this article will be made available by the authors, without undue reservation.
